# Estimates of epidemiology, mortality and disease burden associated with progressive fibrosing interstitial lung disease in France (the PROGRESS study)

**DOI:** 10.1186/s12931-021-01749-1

**Published:** 2021-05-24

**Authors:** Mouhamad Nasser, Sophie Larrieu, Loic Boussel, Salim Si-Mohamed, Fabienne Bazin, Sébastien Marque, Jacques Massol, Françoise Thivolet-Bejui, Lara Chalabreysse, Delphine Maucort-Boulch, Eric Hachulla, Stéphane Jouneau, Katell Le Lay, Vincent Cottin

**Affiliations:** 1 Hôpital Louis Pradel, Centre National de Référence des Maladies Pulmonaires Rares, Hospices Civils de Lyon, Lyon, OrphaLung, RespiFil, ERN-LUNG, Claude Bernard University Lyon 1, 28 Avenue du Doyen Lepine, 69677 Lyon Cedex, France; 2 UMR754, INRAE, Université Claude Bernard Lyon 1, Lyon, France; 3 IQVIA, RWS - La Défense, Paris, France; 4 Département de Radiologie, Hospices Civils de Lyon, Lyon, France; 5 CNRS, Inserm, CREATIS UMR 5220, Université de Lyon, INSA‐Lyon, University Claude Bernard Lyon 1, UJM-Saint Etienne, Lyon, France; 6 AIXIAL, Boulogne-Billancourt, France; 7 Département d’anatomo-pathologie, Hospices Civils de Lyon, Lyon, France; 8 Université de Lyon, 69000 Lyon, France; 9 Université de Lyon 1, 69100 Villeurbanne, France; 10 Service de Biostatistique et Bioinformatique, Hospices Civils de Lyon, Pôle Santé Publique, 69003 Lyon, France; 11 CNRS, UMR 5558, Équipe Biostatistique-Santé, Laboratoire de Biométrie et Biologie Évolutive, 69100 Villeurbanne, France; 12 Service de Médecine Interne et Immunologie Clinique, Hôpital Claude Huriez, Centre National de Référence des maladies auto‑immunes systémiques rare du Nord et Nord‑Ouest de France (CeRAINO), CHU de Lille, Lille, France; 13 Centre Hospitalier Universitaire de Rennes, Centre de Compétences pour les Maladies Pulmonaires Rares, Inserm, EHESP, IRSET (Institut de recherche en santé, environnement et travail), RespiFil, OrphaLung, Univ Rennes, Rennes, France; 14 Boehringer Ingelheim France SAS, Paris, France

**Keywords:** Interstitial lung disease, Progressive fibrosis, Epidemiology, Healthcare resource utilisation, Algorithms

## Abstract

**Background:**

There is a paucity of data on the epidemiology, survival estimates and healthcare resource utilisation and associated costs of patients with progressive fibrosing interstitial lung disease (PF-ILD) in France. An algorithm for extracting claims data was developed to indirectly identify and describe patients with PF-ILD in the French national administrative healthcare database.

**Methods:**

The French healthcare database, the Système National des Données de Santé (SNDS), includes data related to ambulatory care, hospitalisations and death for 98.8% of the population. In this study, algorithms based on age, diagnosis and healthcare consumption were created to identify adult patients with PF-ILD other than idiopathic pulmonary fibrosis between 2010 and 2017. Incidence, prevalence, survival estimates, clinical features and healthcare resource usage and costs were described among patients with PF-ILD.

**Results:**

We identified a total of 14,413 patients with PF-ILD. Almost half of them (48.1%) were female and the mean (± standard deviation) age was 68.4 (± 15.0) years. Between 2010 and 2017, the estimated incidence of PF-ILD ranged from 4.0 to 4.7/100,000 person-years and the estimated prevalence from 6.6 to 19.4/100,000 persons. The main diagnostic categories represented were exposure-related ILD other than hypersensitivity pneumonitis (n = 3486; 24.2%), idiopathic interstitial pneumonia (n = 3113; 21.6%) and rheumatoid arthritis-associated ILD (n = 2521; 17.5%). Median overall survival using Kaplan–Meier estimation was 3.7 years from the start of progression. During the study, 95.2% of patients had ≥ 1 hospitalisation for respiratory care and 34.3% were hospitalised in an intensive care unit. The median (interquartile range) total specific cost per patient during the follow-up period was €25,613 (10,622–54,287) and the median annual cost per patient was €18,362 (6856–52,026), of which €11,784 (3003–42,097) was related to hospitalisations. Limitations included the retrospective design and identification of cases through an algorithm in the absence of chest high-resolution computed tomography scans and pulmonary function tests.

**Conclusions:**

This large, real-world, longitudinal study provides important insights into the characteristics, epidemiology and healthcare resource utilisation and costs associated with PF-ILD in France using a comprehensive and exhaustive database, and provides vital evidence that PF-ILD represents a high burden on both patients and healthcare services.

*Trial registration* ClinicalTrials.gov, NCT03858842. ISRCTN, ISRCTN12345678. Registered 3 January 2019—Retrospectively registered, https://clinicaltrials.gov/ct2/show/NCT03858842

**Supplementary Information:**

The online version contains supplementary material available at 10.1186/s12931-021-01749-1.

## Background

Interstitial lung diseases (ILDs) are a heterogeneous group of disorders [1], which encompass a wide range of conditions [1–3]. In some patients with fibrosing ILDs, a progressive fibrosing phenotype develops comparable to that observed in idiopathic pulmonary fibrosis (IPF), including worsening respiratory symptoms, decline in lung function and early mortality despite standard of care treatment [2, 4, 5]. IPF is the most common and severe progressive fibrosing ILD [5, 6], with established treatments and follow-up strategies [7–9]. However, less evidence exists for patients with other chronic fibrosing ILDs with a progressive phenotype, referred to in this publication as progressive fibrosing ILD (PF-ILD).

The INBUILD^®^ trial recently demonstrated the efficacy of nintedanib, an antifibrotic treatment, to slow down disease progression as assessed by lung function decline, in PF-ILD [10]. Nintedanib has since been approved in the US, and in 45 other countries, including Japan [11], Canada [12] and the European Union for treatment of chronic fibrosing ILDs with a progressive phenotype. Pirfenidone was investigated in patients with progressive unclassifiable ILD [13]. While the study design limited the conclusions that could be drawn from the data, in this study, pirfenidone was observed to decrease the rate of decline in lung function measured in-clinic (a secondary endpoint), indicating that it may benefit patients with progressive fibrosing unclassifiable ILD.

The measure of progression in IPF or PF-ILD in clinical trials is mostly based on progression as defined by forced vital capacity decline, which predicts mortality [14–16]. In clinical practice, patients with progression are therefore more likely to require monitoring with lung function tests [3, 5]. However, monitoring of disease progression includes multiple components, such as symptoms, serial lung function measurements, fibrosis measured by high-resolution computed tomography (HRCT) of the chest, exercise capacity assessments, need for supportive care and, potentially, serum biomarkers [2, 17, 18].

It is expected that healthcare resource consumption will be relatively high in PF-ILD. Healthcare resource consumption may be used as an indirect tool for identifying patients, as there is currently no diagnosis code to identify patients with PF-ILD from medico-administrative databases. Based on insurance claims and survey data in the USA, progressive fibrosis may develop in 18–32% of patients with ILDs other than IPF [19]. In patients with ILDs other than IPF in the USA over a 3-year time frame, ILD-related mean annual medical costs and hospital claims were approximately 75% and 123% higher in patients with a progressive fibrosing phenotype than in patients with non-progressive disease [20].

Although data are available for individual types of PF-ILDs (for example, systemic sclerosis-ILD [SSc-ILD] and other connective tissue disease-ILDs [CTD-ILDs], hypersensitivity pneumonitis [HP], unclassifiable ILD, etc.), data on PF-ILDs as a group come primarily from clinical trials [10, 21] and one academic retrospective series [22]. Therefore, large-scale epidemiological data are limited [20] and this remains an unmet need, as emphasised by a research task force of the American Thoracic Society [23].

The objectives of this study were to assess incidence and prevalence of PF-ILD in France; to characterise patients with PF-ILD at diagnosis; to determine survival rate; and to describe healthcare resource use and associated direct costs of patients with PF-ILD.

## Methods

### Data source and ethics

We conducted a non-interventional, longitudinal, retrospective cohort study using administrative claims data from the French national administrative healthcare database (Système National des Données de Santé [SNDS]), which is managed by the National Health Insurance Fund (Caisse Nationale d’Assurance Maladie [CNAM]).

The SNDS includes, in particular, country-wide health insurance data related to ambulatory care (Système National d'Information Inter-Régimes de l'Assurance Maladie [SNIIRAM]), hospitalisations (Programme de Médicalisation des Systèmes d’Information [PMSI]) and deaths (Centre d’Epidémiologie des Causes de Décès [CépiDc]), and covers 98.8% of the French population of over 66 million people [24]. These merged databases provide comprehensive information on individual activity of healthcare providers, care pathways, healthcare consumption, patient characteristics, hospitalisations and clinical diagnoses in the form of International Classification of Diseases 10th revision (ICD-10) codes.

We used the SNDS database to identify patients with fibrosing ILDs other than IPF, as defined by ICD-10 diagnostic codes appearing on medical claims, and with a progressive phenotype as defined by proxy criteria (see “Case definitions” section).

We then developed an algorithm to select patients with PF-ILD other than IPF within the SNDS using ICD-10 diagnostic codes and healthcare consumption data appearing on medical claims between 1 January 2010 and 31 December 2017.

This study was conducted as part of the wider PROGRESS portfolio under Reference Methodology (MR) 004 [25] following approval by the Comité d’Expertise pour les Recherches, les Etudes et les Evaluations dans le domaine de la Santé (CEREES) on 18 August 2018 (TPS 72584) and by the Commission Nationale de l'Informatique et des Libertés (CNIL) on 9 November 2018 (918305). The PROGRESS study was registered with ClinicalTrials.gov (NCT03858842).

### Case definitions

**Fig. 1:**
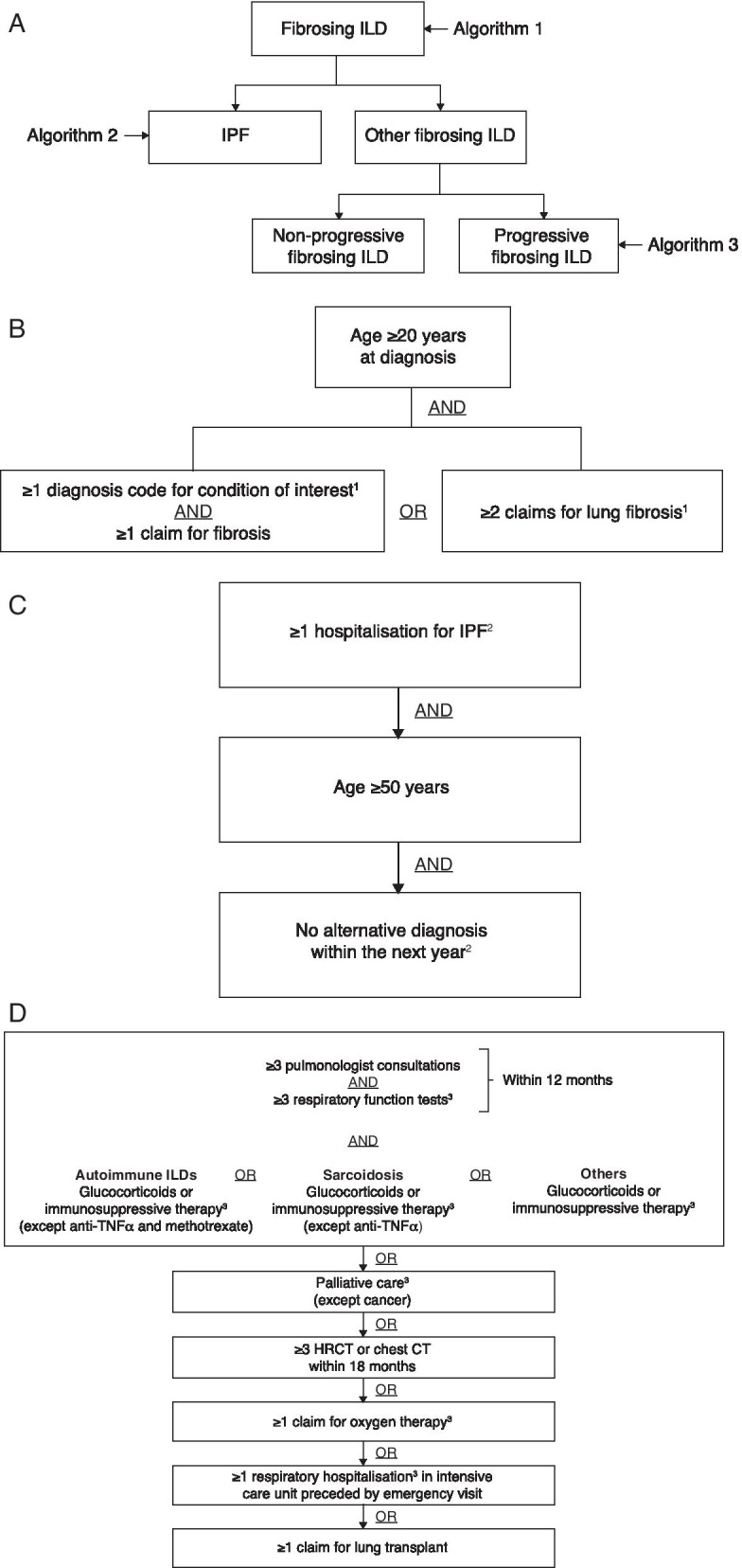
Algorithms for the selection of cases of fibrosing ILD with progressive phenotype from the SNDS. **a** Overview of algorithms and case selection, **b** algorithm 1 for extraction of patients with fibrosing ILD, **c** algorithm 2 for extraction of patients with IPF from the fibrosing ILD group, and **d** algorithm 3 for extraction of patients with fibrosing ILD with a progressive phenotype from the fibrosing ILD group. ^1^For full list of ICD-10 codes and definitions see Additional file 1: Table S1. ^2^For full list of ICD-10 and ATC codes and definitions see Additional file 1: Table S2. ^3^For full list of CCAM, ATC, GHM, LLP and ICD-10 codes and definitions see Additional file 1: Table S3. *ATC* anatomical therapeutic chemical classification, *CCAM* classification commune des actes médicaux [medical classification for clinical procedures], *CT* computed tomography, *GHM* groupes homogènes de maladies [Homogeneous Group of Patients], *HRCT* high-resolution computed tomography, *ICD-X* International Classification of Diseases code, *ILD* interstitial lung disease, *IPF* idiopathic pulmonary fibrosis, *LPP* liste des produits et prestations [list of products and services], *SNDS* Système National des Données de Santé [French national administrative healthcare database], *TNF* tumour necrosis factor.

PF-ILD case definition was based on three algorithms modified from Olson et al. 2020 [20] and developed with clinical experts in France (Fig. 1). Algorithm 1 selected cases with fibrosing ILD with either ≥ 1 claim for one of the qualifying conditions and ≥ 1 claim for fibrosis, or ≥ 2 claims for lung fibrosis. Algorithm 2 was used to identify IPF cases (patients with ≥ 1 claim for hospitalisation for IPF, aged ≥ 50 years and no alternative diagnosis within 12 months for exclusion [i.e. they were not later diagnosed with another fibrosing ILD]; and/or patients receiving antifibrotic treatments at baseline). Algorithm 3 selected cases with fibrosing ILD with a progressive phenotype using proxies for progression (PF-ILD). The proxies for progression required ≥ 3 claims each for pulmonologist consultations and pulmonary function tests (PFTs) within 12 months; and glucocorticoid or immunosuppressive therapy; plus palliative care, or ≥ 3 HRCT or chest computed tomography (CT) scans, or ≥ 1 claim for oxygen therapy, respiratory hospitalisation in an intensive care unit following an emergency visit or lung transplant. The full list of codes used to extract cases is included in Additional file 1: Additional Methods and Tables S1–S3.

### Patient selection

All patients who met the following criteria were included: (1) aged ≥ 20 years; (2) met the criteria for PF-ILD defined above; (3) ≥ 2-year history in the SNDS prior to index date (in order to distinguish between incident and prevalent cases); and (4) affiliated with the general reimbursement scheme. Patients were excluded if they had IPF based on case definitions. The index date was defined as the date of progression, i.e. the first date of a claim that met criteria that are proxies for progression. Patients with a progression before 2010 were considered as having an index date on 1 January 2010, as actual date of progression could not be determined.

### Follow-up period and censoring

The follow-up period was defined from the index date until patient death, end of study period (31 December 2017) or last available record (hospitalisation, consultation or healthcare reimbursement) in the data source, whichever was first. Patients with a data gap persisting beyond 12 months were considered to have ceased follow-up at their last record and were censored at that time.

### Outcomes

Patients’ healthcare resource use was described through the number and percentage of patients who had at least one claim during the follow-up period and, among patients who had at least one, the mean annual number of claims. The following types of healthcare resource use were considered: drugs, other treatments, medical visits (general practitioner, pulmonary specialist, nursing acts, physiotherapy appointments), hospitalisations (all, intensive care unit, acute events and pulmonary hypertension events), laboratory tests, imaging tests, PFTs, ambulance use and sick leave.

Costs were estimated in euros from the national health insurance perspective. For outpatient healthcare resources (general practitioner visits, pulmonary specialist visits, nursing and physiotherapy appointments, laboratory tests, treatments and medical procedures), ambulance use and sick leave, the amount reimbursed by the public healthcare system was directly extracted from the SNDS database. For hospitalisations, the cost of each stay was valued by the diagnosis-related group (Groupe Homogène de Malades [GHM]) using the official tariffs from the French Diagnosis Related Group prospective payment system (source: Agence technique de l’information sur l’hospitalisation, Médecine chirurgie obstétrique et odontologie 2010–2017 tariffs for private and public institutions).

Cost per patient during the follow-up period was calculated as the sum of every healthcare resource use. Annual total costs during the follow-up period were calculated as the sum of every healthcare use divided by the follow-up time. Total cost and annual total cost were calculated by the sum of total costs per patient and the sum of annual total costs per patient.

### Analyses

Descriptive analyses were conducted depending on the criteria. Annual incidence rate was calculated as the proportion of patients who were newly identified as meeting the criteria for PF-ILD (using the three algorithms) during the calendar year of interest (i.e. without any proxy for PF-ILD during the 2 previous years) to all patients at risk (i.e. excluding previously diagnosed cases) aged ≥ 20 years old. Annual prevalence rate was calculated for each year as the proportion of all patients with a proxy for progression during the year of interest to all enrolees who were ≥ 20 years old. Patients contributed to annual incidence only once, but could contribute to prevalence during multiple years. Clopper–Pearson binomial confidence intervals (CIs) were determined for incidence and prevalence.

Characteristics of patients at baseline (date of progression) were described in terms of socio-demographic characteristics and comorbidities for the prevalent cohort. Prevalent comorbidities were defined as those present at baseline or recorded within 1 year prior to the index date. For example, lung cancer reported as a comorbidity in the study could include lung cancer at any time since diagnosis, stage or level of treatment, if present at baseline or recorded within 1 year prior. Mortality rates were calculated in the whole population and for the following subgroups: year (2010–2017), age category and gender. For prevalence and incidence estimates, figures for 2017 would be expected to be lower as two claims were required, and many patients with their first claim in 2017 would not have a second claim before 2018. Therefore, estimates will be presented up to 2016 only. Crude incidence and prevalence rates were calculated in the whole population and for the following subgroups: year (2010–2016), age category, gender and underlying disease.

Overall survival was defined as the time in years from the date of progression to the date of death due to any cause. Overall survival was estimated using the Kaplan–Meier method and subgroups were compared using a logrank test. A multivariable analysis was performed using a Cox’s proportional hazard model, which was checked with Schoenfeld residuals and log cumulative hazard curves based on Kaplan–Meier estimates according to time. All prognostic factors (age, sex, lung cancer, pulmonary hypertension and CTD-ILD) that demonstrated associations with mortality in univariable analyses (p < 0.25) were included in the multivariable model. A manual backward stepwise selection was then used to remove non-significant variables (p ≥ 0.05); between each removed variable, the parameter estimates were checked to avoid deleting a potential confounding factor. All analyses were performed with SAS^®^ for Windows (v 9.4; SAS Institute Inc).

## Results

### Demographic characteristics and comorbidities

**Fig. 2:**
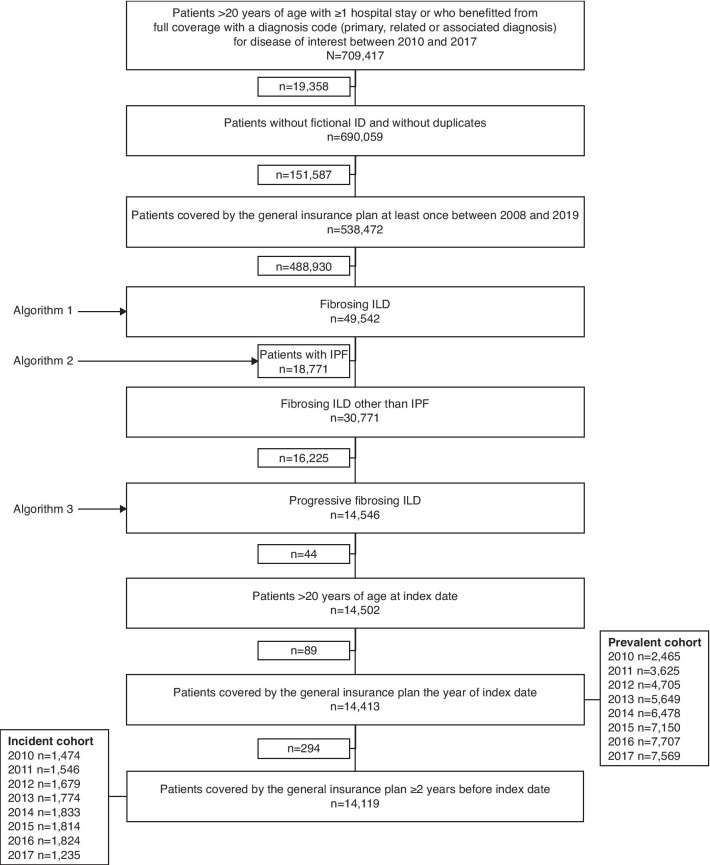
Patient flow chart. *ID* identification, *ILD* interstitial lung disease, *IPF* idiopathic pulmonary fibrosis

Of the 49,542 patients that fitted the definition for fibrosing ILD (Fig. 2), 30,771 patients had a fibrosing ILD other than IPF, and 14,546 of these were predicted to have a progressive phenotype based on the algorithm and will be described using the terminology “patients with PF-ILD”. In total, 14,413 patients met the inclusion criteria and were included in the main analysis (Table 1). Nearly half of patients were female (48.1%). The mean (standard deviation [± SD]) age was 68.4 (± 15.0) years. The median (interquartile range [IQR]) time between diagnosis of fibrosing ILD and meeting PF-ILD criteria was 0.4 years (0.0–4.4). The median (IQR) duration of follow-up was 1.7 years (0.4–3.8). The most frequent diagnoses were exposure-related ILD other than HP (n = 3486; 24.2%), idiopathic interstitial pneumonia (n = 3113; 21.6%) and rheumatoid arthritis-associated ILD (n = 2521; 17.5%). There were also 728 patients (5.1%) with chronic HP.

**Table 1:** Baseline patient characteristics

	PF-ILD (n = 14,413)
Sex, n (%)	
Female	6934 (48.1)
Mean age, years (SD)	68.4 (15.0)
Median duration of follow-up, years (IQR)	1.7 (0.4–3.8)
Subtype of PF-ILD, n (%)	
Exposure-related ILD other than hypersensitivity pneumonitis^a^	3486 (24.2)
Idiopathic interstitial pneumonia	3113 (21.6)
Hypersensitivity pneumonitis	728 (5.1)
Autoimmune ILD	
RA-ILD	2521 (17.5)
SSc-ILD	907 (6.3)
MCTD-ILD	655 (4.5)
Other autoimmune^b^	1503 (10.4)
Sarcoidosis-ILD	1500 (10.4)

Baseline was defined as the date of progression

*ILD* interstitial lung disease, *IQR* interquartile range, *MCTD* mixed connective tissue disease, *PF-ILD* progressive fibrosing interstitial lung disease, *RA* rheumatoid arthritis, *SD* standard deviation, *SSc* systemic sclerosis

^a^Coal workers’ pneumoconiosis (n = 144), asbestosis (n = 878), pneumoconiosis due to other dust containing silica (n = 399), aluminosis of the lung (n = 1), bauxite fibrosis of the lung (n = 13), berylliosis (n = 8), graphite fibrosis of the lung (n = 28), siderosis (n = 25), pneumoconiosis due to other specified inorganic dusts (n = 19), unspecified pneumoconiosis (n = 130), byssinosis (n = 2), cannabinosis (n = 1), bronchitis and pneumonitis due to chemicals, gases, fumes and vapours (n = 42), chronic respiratory conditions due to chemicals, gases, fumes and vapours (n = 119), chronic and other pulmonary manifestations due to radiation (n = 450), chronic drug-induced interstitial lung disorders (n = 454), unspecified drug-induced interstitial lung disorders (n = 638), respiratory conditions due to other specified external agents (n = 46), respiratory conditions due to unspecified external agent (n = 89)

^b^Sjogren syndrome (n = 804), polymyositis (n = 435) and systemic lupus erythematosus (n = 264)

The majority of patients were affected by comorbidities at baseline, the most common of which were hypertension (63.8%) and gastroesophageal reflux disease (55.4%) (Table 2).

**Table 2:** Comorbidities at baseline

Comorbidities at baseline, n (%)	PF-ILD (n = 14,413)
Arterial hypertension	9193 (63.8)
Gastroesophageal reflux disease	7991 (55.4)
Cardiac arrhythmias	3155 (21.9)
Depression	2953 (20.5)
Congestive heart failure	2886 (20.0)
Chronic coronary disease	2227 (15.5)
Lung cancer	940 (6.5)
Anaemia	826 (5.7)
Pulmonary hypertension	765 (5.3)
Diarrhoea	486 (3.4)
Digital ulcer	475 (3.3)
Osteoporosis	399 (2.8)
Acute coronary syndrome	284 (2.0)
Cirrhosis	172 (1.2)

Baseline was defined as the date of progression

*PF-ILD* progressive fibrosing interstitial lung disease

### Prevalence and incidence of PF-ILD

There were 14,413 patients identified in the prevalent cohort and 14,119 in the incident cohort (Additional file 1: Table S4). The overall incidence estimates for PF-ILD per 100,000 person-years ranged from 4.0 to 4.7. Incident rates were numerically higher among men versus women. The overall prevalence estimates for PF-ILD per 100,000 persons increased each year from 6.6 to 19.4 in 2016. Prevalence was numerically higher amongst men versus women (Additional file 1: Table S4).

### Survival in patients with PF-ILD

**Fig. 3:**
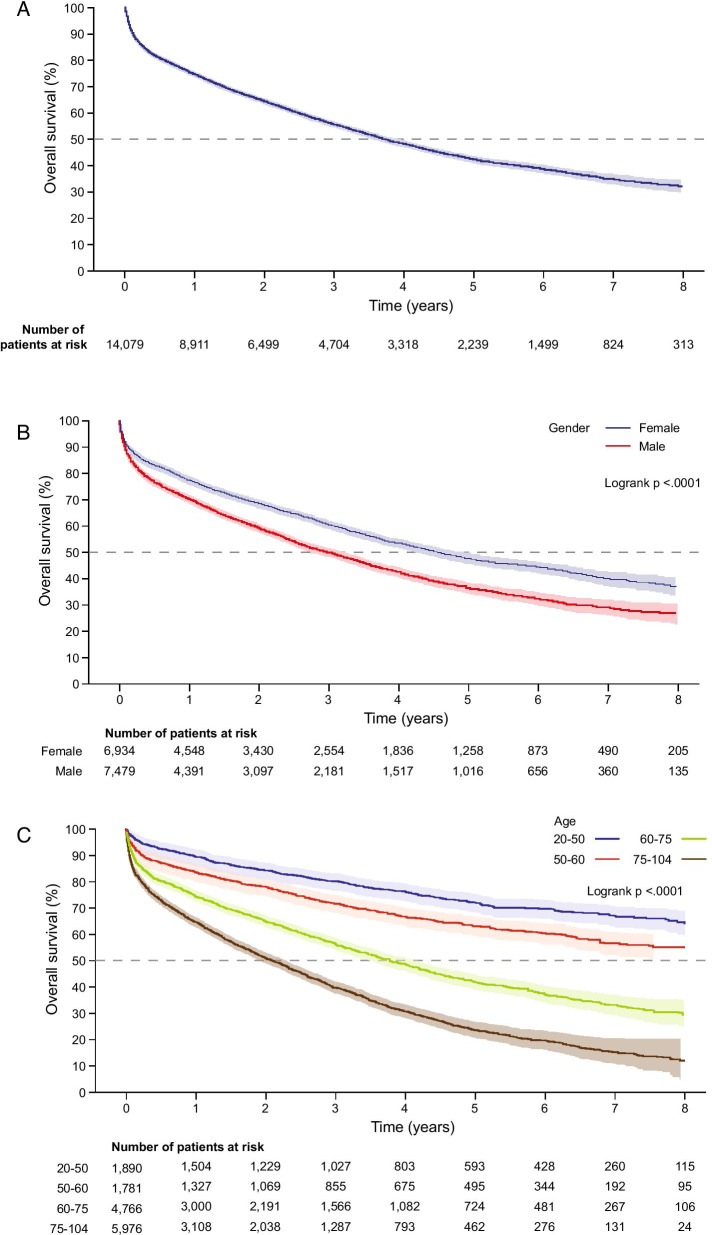

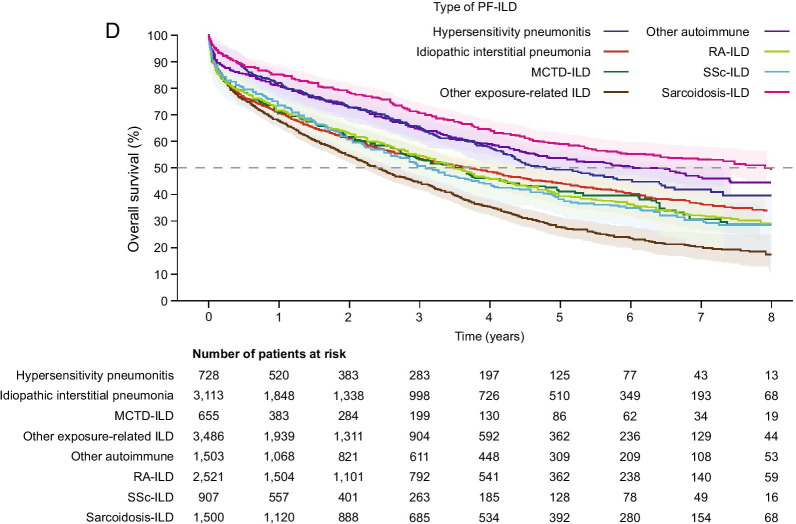
Overall survival for PF-ILD. **a** Overall survival among all patients, and by **b** sex, **c** age, and **d** diagnosis subgroup. Overall survival was defined as the time in years from the date of progression to the date of death due to any cause. Shading indicates 95% Hall–Wellner band. *ILD* interstitial lung disease, *MCTD* mixed connective tissue disease, *PF-ILD* progressive fibrosing interstitial lung disease, *RA* rheumatoid arthritis, *SSc* systemic sclerosis

In total, 6096 (42.3%) patients were still alive at the end of follow-up; 1537 (10.7%) were lost to follow-up or censored, and 6780 (47.0%) had died. Median overall survival from the beginning of progression was 3.7 years (95% CI 3.6–3.8) (Fig. 3A). Median overall survival was longer in females compared with males (4.6 vs 3.0 years) (Fig. 3B; Additional file 1: Table S5), and older age groups showed a marked decline in overall survival (Fig. 3C; Additional file 1: Table S6). When split by underlying disease, patients with sarcoidosis-ILD had the longest median overall survival (7.9 years) and patients with exposure-related ILD other than HP had the shortest (2.4 years) (Fig. 3D; Additional file 1: Table S7).

The crude multivariable Cox model found male gender, age categories ≥ 50 years and underlying disease to be significantly associated with mortality (all p < 0.0001; Additional file 1: Table S8). A check of the proportional hazards assumption for the factors selected for the multivariable analysis found a significant interaction for time with age and underlying disease (both p < 0.0001).

For the multivariable analysis, different models were produced based on year of progression as the time × subgroup diagnosis interaction was significant. As shown in Fig. 3D, the curves overlap during the first year; therefore, 1 year was chosen as a cut-off point. Two final Cox models were built: (1) factors associated with mortality in the first year post-progression, and (2) factors associated with mortality after the first year post-progression. Model 1 (first year post-progression, n = 14,413) confirmed male sex, age categories ≥ 50 years and underlying disease to be significantly associated with mortality in the first year post-progression (all p < 0.0001; Table 3). HP, other autoimmune and sarcoidosis-ILD had the lowest mortality risk. Model 2 (after first year post-progression, n = 8928) found that male sex (p < 0.0001), age categories ≥ 50 years (p < 0.0001) and underlying disease (p < 0.0001) were significantly associated with mortality after the first year post-progression (Table 3). Exposure-related ILDs other than HP (p = 0.0015) and SSc-ILD (p < 0.0001) had a significantly higher mortality risk than HP. There was no difference in the findings of the two models.

**Table 3:** Final multivariable Cox model of factors associated with mortality

Parameter	Category	HR (95% CI)	p-value	Type 3 test p-value
Model 1. Factors associated with mortality in the first year post-progression (n = 14,413)				
Sex	Female	1.00		< 0.0001
	Male	1.32 (1.23–1.42)	< 0.0001	
Categorised age^a^	≥ 20–< 50	1.00		< 0.0001
	≥ 50–< 60	1.74 (1.44–2.09)	< 0.0001	
	≥ 60–< 75	2.65 (2.27–3.10)	< 0.0001	
	≥ 75–< 104	3.87 (3.33–4.51)	< 0.0001	
Underlying disease^b^	HP	1.00		< 0.0001
	Sarcoidosis-ILD	1.00 (0.80–1.25)	0.9916	
	Other autoimmune^c^	1.23 (0.99–1.52)	0.0569	
	RA-ILD	1.53 (1.26–1.86)	< 0.0001	
	Exposure-related ILD other than HP^d^	1.57 (1.30–1.89)	< 0.0001	
	MCTD-ILD	1.62 (1.29–2.04)	< 0.0001	
	SSc-ILD	1.76 (1.42–2.20)	< 0.0001	
	IIP	1.92 (1.59–2.32)	< 0.0001	
Model 2. Factors associated with mortality after the first year post-progression (n = 8928)^e^				
Sex	Female	1.00		< 0.0001
	Male	1.32 (1.23–1.42)	< 0.0001	
Categorised age^a^	≥ 20–< 50	1.00		< 0.0001
	≥ 50–< 60	1.31 (1.11–1.55)	0.0015	
	≥ 60–< 75	2.58 (2.25–2.96)	< 0.0001	
	≥ 75–< 104	4.43 (3.87–5.08)	< 0.0001	
Underlying disease^b^	HP	1.00		< 0.0001
	Sarcoidosis-ILD	0.94 (0.78–1.15)	0.5603	
	Other autoimmune^c^	0.97 (0.80–1.18)	0.7828	
	RA-ILD	1.03 (0.86–1.23)	0.7455	
	MCTD-ILD	1.08 (0.86–1.36)	0.4861	
	IIP	1.13 (0.95–1.35)	0.1608	
	Exposure-related ILD other than HP^d^	1.32 (1.11–1.56)	0.0015	
	SSc-ILD	1.64 (1.34–2.01)	< 0.0001	

And for these models, the proportional hazards assumption was respected

*CI* confidence interval, *HR* hazard ratio, *HP* hypersensitivity pneumonitis, *IIP* idiopathic interstitial pneumonia, *ILD* interstitial lung disease, *MCTD* mixed connective tissue disease, *PF-ILD* progressive fibrosing interstitial lung disease, *RA* rheumatoid arthritis, *SSc* systemic sclerosis

^a^Age categories were each compared with the 20–50 years age group

^b^Underlying diseases were each compared with the hypersensitivity pneumonitis subgroup

^c^Sjogren syndrome, polymyositis and systemic lupus erythematosus

^d^Coal workers’ pneumoconiosis, asbestosis, pneumoconiosis due to other dust containing silica, aluminosis of the lung, bauxite fibrosis of the lung, berylliosis, graphite fibrosis of the lung, siderosis, pneumoconiosis due to other specified inorganic dusts, unspecified pneumoconiosis, byssinosis, cannabinosis, bronchitis and pneumonitis due to chemicals, gases, fumes and vapours, chronic respiratory conditions due to chemicals, gases, fumes and vapours, chronic and other pulmonary manifestations due to radiation, chronic drug-induced interstitial lung disorders, unspecified drug-induced interstitial lung disorders, respiratory conditions due to other specified external agents, respiratory conditions due to unspecified external agent

^e^Patients who were still followed 1 year after progression

### Healthcare resource utilisation and cost evaluation

Regardless of diagnosis, patients received glucocorticoids as their primary therapy: > 60% in each underlying disease and 68.5% overall. The other most commonly used drug treatments were mycophenolate mofetil (MMF) or mycophenolic acid (7.0%) and azathioprine (6.7%); 6020 (41.8%) patients also received supplemental oxygen therapy (Table 4).

**Table 4:** Treatment during follow-up

	PF-ILD (n = 14,413)	
	Patients with ≥ 1, n (%)	Median annual, n (IQR)^a^
Drug consumption		
Glucocorticoids (IV or oral)	9871 (68.5)	7.3 (2.2–11.0)
Mycophenolate mofetil or mycophenolic acid	1007 (7.0)	5.0 (2.0–8.1)
Azathioprine	960 (6.7)	3.0 (1.0–5.9)
Methotrexate	461 (3.2)	1.9 (0.6–5.0)
Rituximab	355 (2.5)	0.9 (0.5–1.6)
Cyclophosphamide	67 (0.5)	1.5 (0.6–3.0)
Anti-TNFα	239 (1.7)	5.4 (1.6–9.0)
Tocilizumab	27 (0.2)	1.8 (0.6–5.3)
Antifibrotics	229 (1.6)	3.4 (1.5–7.6)
Other treatment		
Supplemental oxygen use	6020 (41.8)	9.2 (4.2–13.0)
Lung transplantation	126 (0.9)	0.2 (0.2–0.5)
Palliative care	3229 (22.4)	4.4 (0.8–17.2)
Haematopoietic stem cell transplantation	60 (0.4)	0.4 (0.2–0.8)

End of follow-up was defined as the earliest of patient death, end of study period (31 December 2017) or last available record (hospitalisation, consultation or healthcare reimbursement) in the data source. Patients with a data gap persisting beyond 12 months are considered to have their follow-up ceased at their last record

*IQR* interquartile range, *IV* intravenous, *PF-ILD* progressive fibrosing interstitial lung disease, *TNF* tumour necrosis factor

^a^Median annual value for those with ≥ 1 claim during the study period

In total, 13,727 (95.2%) patients had at least one hospitalisation during the follow-up period, with an annual median (IQR) hospitalisation rate of 3.9 (1.7–9.5) per year (Table 5). A total of 75.2% of patients had a hospitalisation due to acute events, 11.0% were hospitalised for pulmonary hypertension and 34.3% were in an intensive care unit. Regarding imaging, 89.2% of patients had pulmonary imaging, including 85.0% who had a chest X-ray and 69.2% who had a chest HRCT scan.

**Table 5:** Healthcare resource utilisation during follow-up

	PF-ILD (n = 14,413)	
	Patients with ≥ 1, n (%)	Median annual, n (IQR)^a^
Medical visits		
General practitioners visits	12,476 (86.6)	11.2 (6.9–17.4)
Pulmonary specialist visits	8870 (61.5)	2.1 (1.1–3.9)
Nursing acts	11,301 (78.4)	17.6 (5.1–65.6)
Physiotherapy acts	7982 (55.4)	17.5 (6.1–50.5)
Hospitalisations		
All-cause hospitalisation	13,727 (95.2)	3.9 (1.7–9.5)
Acute event hospitalisation	10,835 (75.2)	1.8 (0.7–4.8)
Pulmonary hypertension hospitalisation	1591 (11.0)	0.9 (0.4–2.1)
ICU	4944 (34.3)	0.8 (0.4–2.4)
Ambulance use	12,176 (84.5)	7.8 (3.1–16.1)
Sick leave daily allowances	1630 (11.3)	9.2 (3.7–19.5)
Laboratory analyses	11,293 (78.4)	12.2 (5.9–24.5)
Pulmonary function tests	10,670 (74.0)	3.1 (1.6–5.7)
Imaging		
Pulmonary imaging	12,858 (89.2)	3.9 (2.1–7.8)
Chest X-ray	12,258 (85.0)	2.6 (1.3–5.6)
Chest or body scan	9971 (69.2)	1.4 (0.7–2.6)

End of follow-up was defined as the earliest of patient death, end of study period (31 December 2017) or last available record (hospitalisation, consultation or healthcare reimbursement) in the data source. Patients with a data gap persisting beyond 12 months are considered to have their follow-up ceased at their last record

*ICU* intensive care unit, *IQR* interquartile range, *PF-ILD* progressive fibrosing interstitial lung disease

^a^Median annual value for those with ≥ 1 claim during the study period

The median (IQR) total specific cost per patient during follow-up was €25,613 (10,622–54,287) and the median annual cost per patient was €18,362 (6856–52,026) (Table 6). The total median annual costs (IQR) per patient for all hospitalisations, specific laboratory tests, imagery and treatment were €11,784 (3003–42,097), €39 (7–93), €37 (0–111) and €17 (0–73), respectively. The total median (IQR) specific medical and paramedical cost per patient (excluding sick leave, daily allowances and transport costs) was €1634 (344–4451), and the annual cost per patient was €868 (345–2333).

**Table 6:** Total specific costs during follow-up

	PF-ILD (n = 14,413)	
	Mean, € (SD)	Median, € (IQR)
Cost per patient	43,807 (57,904)	25,613 (10,622–54,287)
Annual cost per patient	81,286 (21,935)	18,362 (6,856–52,026)

End of follow-up was defined as the earliest of patient death, end of study period (31 December 2017) or last available record (hospitalisation, consultation or healthcare reimbursement) in the data source. Patients with a data gap persisting beyond 12 months are considered to have their follow-up ceased at their last record. Costs were based on non-hospital medical procedures (CCAM). Respiratory tests performed during a hospital stay were not taken into account contrary to indicators

*CCAM* classification commune des actes médicaux [medical classification for clinical procedures], *IQR* interquartile range, *PF-ILD* progressive fibrosing interstitial lung disease, *SD* standard deviation

## Discussion

This study investigated the epidemiology, clinical characteristics, mortality and healthcare resource use and associated costs of patients meeting criteria for PF-ILD in France. The data presented are estimates based on an algorithm to identify patients with a progressive phenotype among a fibrotic ILD population. In addition, this is one of the first studies presenting information on PF-ILDs as a whole based on such a comprehensive national-level database, with an estimated 14,413 patients. A previous US claims-based study was based on 373 patients with PF-ILD [20].

The percentage of patients with fibrosing ILDs other than IPF that developed progression was estimated at 18–32% in a survey of pulmonary, rheumatology and internal medicine physicians from Japan, the US and four European countries [19], and at 15% in a US claims database study [20]. We have previously described a single-centre clinical cohort with PF-ILD as part of the PROGRESS study [22] where, similarly, 27.2% of patients with a fibrosing ILD other than IPF had a progressive phenotype. In the current study, approximately 47% of patients with fibrosing ILD other than IPF had a progressive phenotype. This higher percentage of patients with a progressive phenotype may be due to the fact that patients were tentatively identified based on high consumption of respiratory healthcare in a limited period of time, rather than just the results of sequential PFTs, chest CT and symptoms [22] or a physician’s survey [19]. In a preliminary validation study, algorithm 3 was found to have a specificity of 69% and a sensitivity of 46% (data not shown), indicating that our estimates were conservative. While both the current and the US study [20] used healthcare claims to identify progression, differences between these studies may be reflective of how patients in France and the US access care and reimbursement and regulatory systems. There was a high level of comorbidities, with most patients experiencing one or more, particularly hypertension and gastroesophageal reflux disease. This may indicate a sicker patient population with a higher rate of progression than in other studies. However, these comorbidities, and the individual entities comprising the group of PF-ILDs, were consistent with the characteristics of the PROGRESS clinical cohort [22].

No direct comparison was performed in the current study between patients with IPF and those with PF-ILD other than IPF. However, in a previous study of hospitalised patients with IPF in France (n = 6476), the most frequently reported comorbidities were chronic respiratory insufficiency (25.0%), chronic obstructive pulmonary disease (17.6%) and heart failure (17.4%) [26]; the in-hospital mortality rate at 5 years after first hospitalisation was 43.0%. In comparison, we found that the most frequent comorbidities in patients with PF-ILD were arterial hypertension (63.8%), gastroesophageal reflux disease (55.4%) and cardiac arrhythmias (21.9%), with overall survival at 5 years of 42.0%.

There was a trend for increased prevalence and incidence rates of PF-ILD from 2010 to 2016. As seen in the absolute cohort numbers, there were fewer patients in the prevalent and incident cohorts in 2017. In 2017, the incidence is likely to be under-represented due to non-identification of cases where a patient with one claim in 2017 can only be identified as having progressed with a second claim after the study end date. For this reason, we consider that the best estimates of incidence and prevalence are those for the 2015–2016 period. Our study was not designed to track changes in prevalence and incidence over time, and therefore trends should be interpreted with caution due to possible artefacts in the methodology and/or algorithm.

This study, based on national real-world claims data, confirms that PF-ILD is linked to poor prognosis and high mortality [5, 22]. Furthermore, median overall survival was 3.7 years, with an estimated 31.6% of patients surviving at the end of 8 years’ follow-up (mean age at baseline 68.4 years), which is consistent with the high level of comorbidities and progression observed. In our clinical cohort, mortality rates were lower and median overall survival was not reached, with an estimated 65% of patients surviving at 7 years’ follow-up (mean age at baseline 61 years) [22]. The difference may be due to referral bias, with our clinical cohort being drawn from a single expert tertiary reference centre where patients may receive more specialist care than the general population covered by the SNDS.

Factors associated with increased mortality included male gender, age ≥ 50 years and underlying disease subgroup, especially those with SSc-ILD or other exposure. In the PROGRESS clinical cohort [22], age ≥ 50 years and underlying disease (HP, idiopathic interstitial pneumonia and unclassifiable ILD) were also significantly associated with mortality. The current study identified many of the same underlying disease subgroups and associated them with decreased overall survival.

There are currently no treatment guidelines for PF-ILDs as a whole, although recommendations do exist for SSc-ILD [27, 28]. Treatment with cyclophosphamide is recommended for patients with SSc-ILD, or haematopoietic stem cell transplant for selected patients; nevertheless, these treatments were only reported for 1.3% and 0.8% of patients with that diagnosis in our study. MMF is increasingly used in patients with SSc-ILD or chronic HP, but we identified prescription claims in only 7% of patients. However, it should be noted that prescription claims are only recorded for outpatients in the SNDS; therefore, prescriptions for intravenous cyclophosphamide or rituximab, for example, may be underestimated as they are administered only in a hospital setting, whereas MMF can be administered at home. Less than 2% of patients had at least one claim for antifibrotic treatment during the study. This would have been off-label use as nintedanib has not yet been approved for use in patients with PF-ILD in France, and was only approved in the EU for treatment of other chronic fibrosing ILDs with a progressive phenotype in mid-2020. Immunosuppressive drugs are the main therapy for CTD-ILD [29], as seen with mixed CTD-ILD in the current study. Regardless of diagnosis, patients received glucocorticoids as their primary therapy: more than 60% in each underlying disease. Similarly, a US claims database study found that 49–69% of patients with PF-ILDs received glucocorticoids [19].

For patients with IPF, after adjusting for cost years and currency, the estimated mean total annual cost per capita in North America is $20,000, approximately three times higher than per capita healthcare expenditure for the general population [30]. In a US study of PF-ILD between 2014 and 2016, mean annual medical costs per patient were $77,666, and were $35,364 for ILD-specific claims [20], with 83.6% of the medical costs associated with hospital claims [20]. Similarly, in our study, the total mean annual cost per patient with PF-ILD was €81,286, and for all hospitalisations was €54,679 per patient. Most patients were hospitalised at least once during the study period, with 34.3% having at least one admission to intensive care, demonstrating the high burden of disease on patients. We estimate our data to represent the cost to the French National Health Insurance Fund, and that there would be negligible co-payments by patients, with an estimated 79.2% of patients in the study having full health coverage.

Overall, healthcare resource data for investigations and treatments suggest that patients with PF-ILD may not receive sufficient care, and may benefit from being treated in specialised centres.

Limitations of this study include the absence of PFT and imaging data, which precludes individual assessment of disease progression; however, this study provides a macroscopic view of PF-ILD and is complementary to our clinical cohort study, which did include such data [22]. Inclusion criteria were dependent on physicians accurately assigning diagnostic codes, leading to the possibility of miscoding. As there is no specific code for PF-ILD, it is difficult to identify patients using healthcare system data and depends on the accuracy of each part of the algorithmic stepwise approach. For example, the number of patients diagnosed with hypersensitivity pneumonitis was lower than that of other exposure-related ILD, and there was a higher proportion of patients with pneumoconiosis (25%) than reported in epidemiology studies [31]. We cannot exclude the possibility that cases may have been miscoded, or that patients may have been originally coded as pneumoconiosis based on some exposure to asbestos, silica, etc. before a different diagnosis was confirmed. Coding systems may also change over time, partly due to modifications to suit reimbursement purposes. Prevalence and incidence could be underestimated because only patients hospitalised or with full coverage were included; this would exclude any patients who were followed up as outpatients and not hospitalised at any time during the 2010–2017 period. However, the scope and completeness of the SNDS database, with the linkage within SNIIRAM between PMSI (hospitalisation), CepiDC (death), and CNAM (national security data for reimbursement of ambulatory care), allowed us to estimate the national incidence and prevalence rates of this patient population. This study evaluated the overall survival rate but was not able to assess the causes of death in PF-ILD. However, use of supplemental oxygen in 41.8% of patients suggests that they developed chronic respiratory failure, and respiratory causes were the primary causes of death in our clinical cohort [22].

In conclusion, this real-world study provides important insights into the clinical characteristics, epidemiology and healthcare resource utilisation and costs associated with PF-ILD in France, and provides vital evidence that PF-ILD represents a high burden on both patients and healthcare services.

## Supplementary Information

**Additional file 1.** Additional methods and results.

## Data Availability

The data are publicly available on request from the SNDS.

## Abbreviations

CEREES: Comité d’Expertise pour les Recherches, les Etudes et les Evaluations dans le domaine de la Santé
CépiDc: Centre d’épidémiologie des causes de décès
CI: Confidence interval
CNAM: Caisse Nationale d'Assurance Maladie
CNIL: Commission Nationale de l'Informatique et des Libertés
CT: Computed tomography
CTD-ILD: Connective tissue disease-ILD
GHM: Groupe Homogène de Malade
HP: Hypersensitivity pneumonitis
HRCT: High-resolution computed tomography
ICD: International Classification of Diseases
ILD: Interstitial lung disease
IPF: Idiopathic pulmonary fibrosis
IQR: Interquartile range
MMF: Mycophenolate mofetil
PF-ILD: Progressive fibrosing interstitial lung disease
PFT: Pulmonary function test
PMSI: Programme de médicalisation des systèmes d’information
SD: Standard deviation
SNDS: Système National des Données de Santé
SNIIRAM: Système national d'information inter-régimes de l'Assurance maladie
SSc-ILD: Systemic sclerosis-ILD
