# Supplementary material for: Estimates of epidemiology, mortality and disease burden associated with progressive fibrosing interstitial lung disease in France (the PROGRESS study)
Source: Respir Res. 2021 May 24;22:162. doi: 10.1186/s12931-021-01749-1 (PMC8147348; doi:10.1186/s12931-021-01749-1)
Supplement: Supplementary file 1 — Additional file 1. Additional methods and results. [file 12931_2021_1749_MOESM1_ESM.docx]

**Supplement**

**Estimates of epidemiology, mortality and disease burden associated with progressive fibrosing interstitial lung disease in France (the PROGRESS study)**

Mouhamad Nasser^1,2^ (ORCiD 0000-0001-8373-8032), Sophie Larrieu^3^, Loic Boussel^4^, Salim Si-Mohamed^4,5^, Fabienne Bazin^3^, Sébastien Marque^3^, Jacques Massol^6^, Franҫoise Thivolet-Bejui^7^, Lara Chalabreysse^7^, Delphine Maucort-Boulch^8^, Eric Hachulla^10^, Stéphane Jouneau^1,9^, Katell Le Lay^11^, Vincent Cottin^1,2^

^1^Hôpital Louis Pradel, Centre National de Référence des Maladies Pulmonaires Rares, Hospices Civils de Lyon, Lyon, OrphaLung, RespiFil, ERN-LUNG, France; ^2^Université Claude Bernard Lyon 1, UMR754, INRAE, Lyon, France; ^3^IQVIA, RWS – La Défense, Paris, France; ^4^Département de Radiologie, Hospices Civils de Lyon, Lyon, France; ^5^Université de Lyon, INSA‐Lyon, University Claude Bernard Lyon 1, UJM-Saint Etienne, CNRS, Inserm, CREATIS UMR 5220, Lyon, France; ^6^AIXIAL – Boulogne-Billancourt, France; ^7^Département d’anatomo-pathologie, Hospices Civils de Lyon, Lyon, France; ^8^Université de Lyon, F-69000, Lyon, France; Université Lyon 1, F-69100, Villeurbanne, France; Hospices Civils de Lyon, Pôle Santé Publique, Service de Biostatistique et Bioinformatique, F-69003, Lyon, France; CNRS, UMR 5558, Laboratoire de Biométrie et Biologie Évolutive, Équipe Biostatistique-Santé, F-69100, Villeurbanne, France; ^9^Centre Hospitalier Universitaire de Rennes, Centre de Compétences pour les Maladies Pulmonaires Rares, Univ Rennes, Inserm, EHESP, IRSET (Institut de recherche en santé, environnement et travail), RespiFil, OrphaLung, Rennes, France; ^10^Service de Médecine Interne et Immunologie Clinique, Hôpital Claude Huriez, Centre National de Référence des maladies auto-immunes systémiques rare du Nord et Nord-Ouest de France (CeRAINO), CHU de Lille, Lille, France; ^11^Boehringer Ingelheim France SAS, Paris, France

**Supplementary Methods**

**Algorithm 1**

- Algorithm 1 was used to detect patients with fibrosing ILD. The ICD-10 codes for pathologies of interest and pulmonary fibrosis are in Table S1.

**Algorithm 2**

- Algorithm 2 was used to detect patients with IPF in order to exclude them from the analysis. The ICD-10 codes for IPF, fibrosing ILD and differential diagnoses, and the ATC codes for antifibrotic treatment are in Table S2.

**Algorithm 3**

- Algorithm 3 was used to detect patients with progressive fibrosing ILD. The ATC codes for glucocorticoids and immunosuppressant treatment, the CCAM codes for imaging and respiratory function tests, the LLP and ICD-10 codes for oxygen therapy, the GHM and ICD-10 codes for palliative care, and the ICD-10 codes for hospitalisations for respiratory problems are in Table S3. The date of progression was the first date of precedent event.

**Table S1. Identification codes for algorithm 1**

| **ICD-10 code** | **Pathologies of interest** |
| --- | --- |
| **Sarcoidosis** | |
| D860 | Sarcoidosis of lung |
| D861 | Sarcoidosis of lymph nodes |
| D862 | Sarcoidosis of lung with sarcoidosis of lymph nodes |
| D863 | Sarcoidosis of skin |
| D868 | Sarcoidosis of other and combined sites |
| D869 | Sarcoidosis, unspecified |
| **Lung disease due to external agents** | |
| J60 | Coal workers' pneumoconiosis |
| J61 | Asbestosis |
| J628 | Pneumoconiosis due to other dust containing silica |
| J630 | Aluminosis (of lung) |
| J631 | Bauxite fibrosis (of lung) |
| J632 | Berylliosis |
| J633 | Graphite fibrosis (of lung) |
| J634 | Siderosis |
| J635 | Stannosis |
| J638 | Pneumoconiosis due to other specified inorganic dusts |
| J64 | Unspecified pneumoconiosis |
| J660 | Byssinosis |
| J661 | Flax-dressers' disease |
| J662 | Cannabinosis |
| J670 | Farmer lung |
| J671 | Bagassosis |
| J672 | Bird fancier lung |
| J673 | Suberosis |
| J674 | Maltworker lung |
| J675 | Mushroom-worker lung |
| J676 | Maple-bark-stripper lung |
| J677 | Air-conditioner and humidifier lung |
| J678 | Hypersensitivity pneumonitis due to other organic dusts |
| J679 | Hypersensitivity pneumonitis due to unspecified organic dust |
| J680 | Bronchitis and pneumonitis due to chemicals, gases, fumes and vapours |
| J684 | Chronic respiratory conditions due to chemicals, gases, fumes and vapours |
| J701 | Chronic and other pulmonary manifestations due to radiation |
| J703 | Chronic drug-induced interstitial lung disorders |
| J704 | Drug-induced interstitial lung disorders, unspecified |
| J708 | Respiratory conditions due to other specified external agents |
| J709 | Respiratory conditions due to unspecified external agent |
| **Rheumatoid arthritis and other inflammatory polyarthropathies** | |
| M050 | Felty's syndrome, unspecified site |
| M051 | Rheumatoid lung disease |
| M052 | Rheumatoid vasculitis |
| M053 | Rheumatoid arthritis with involvement of other organs and systems |
| M061 | Adult-onset Still's disease |
| M064 | Inflammatory polyarthropathy |
| M068 | Other specified rheumatoid arthritis |
| M069 | Rheumatoid arthritis, unspecified |
| M120 | Chronic postrheumatic arthropathy |
| M130 | Polyarthritis, unspecified |
| **Systemic connective tissue disorder** | |
| M321 | Systemic lupus erythematosus, organ or system involvement unspecified |
| M328 | Other forms of systemic lupus erythematosus |
| M329 | Systemic lupus erythematosus, unspecified |
| M331 | Other dermatomyositis |
| M332 | Polymyositis |
| M339 | Dermatopolymyositis, unspecified |
| M340 | Progressive systemic sclerosis |
| M341 | CR(E)ST syndrome |
| M342 | Systemic scleroderma due to drugs and chemicals |
| M348 | Other forms of systemic sclerosis |
| M349 | Systemic sclerosis, unspecified |
| M350 | Sicca syndrome [Sjögren] |
| M351 | Other overlap syndromes |
| M352 | Behçet disease |
| M353 | Polymyalgia rheumatica |
| M354 | Diffuse (eosinophilic) fasciitis |
| M355 | Multifocal fibrosclerosis |
| M356 | Relapsing panniculitis |
| M357 | Hypermobility syndrome |
| M358 | Other specified systemic involvement of connective tissue |
| M359 | Systemic involvement of connective tissue, unspecified |
| **Lung fibrosis** | |
| J178 | Pneumonia in other diseases classified elsewhere |
| J990 | Rheumatoid lung disease |
| J840 | Alveolar and parietoalveolar conditions |
| J841 | Other interstitial pulmonary diseases with fibrosis |
| J848 | Other specified interstitial pulmonary diseases |
| J849 | Interstitial pulmonary disease, unspecified |

ICD-10, International Classification of Diseases 10^th^ revision.

**Table S2. Identification codes for algorithm 2**

| **ATC code** | **Antifibrotic treatment** |
| --- | --- |
| L01XE31 | Nintedanib |
| L04AX05 | Pirfenidone |
| **ICD-10 code** | **Idiopathic pulmonary fibrosis** |
| J84.1 | Other interstitial pulmonary diseases with fibrosis |
| **ICD-10 code** | **Differential diagnoses** |
| **Sarcoidosis** | |
| D860 | Sarcoidosis of lung |
| D861 | Sarcoidosis of lymph nodes |
| D862 | Sarcoidosis of lung with sarcoidosis of lymph nodes |
| D863 | Sarcoidosis of skin |
| D868 | Sarcoidosis of other and combined sites |
| D869 | Sarcoidosis, unspecified |
| **Lung disease due to external agents** | |
| J60 | Coal workers' pneumoconiosis |
| J61 | Asbestosis |
| J628 | Pneumoconiosis due to other dust containing silica |
| J630 | Aluminosis (of lung) |
| J631 | Bauxite fibrosis (of lung) |
| J632 | Berylliosis |
| J633 | Graphite fibrosis (of lung) |
| J634 | Siderosis |
| J635 | Stannosis |
| J638 | Pneumoconiosis due to other specified inorganic dusts |
| J64 | Unspecified pneumoconiosis |
| J660 | Byssinosis |
| J661 | Flax-dressers' disease |
| J662 | Cannabinosis |
| J670 | Farmer lung |
| J671 | Bagassosis |
| J672 | Bird fancier lung |
| J673 | Suberosis |
| J674 | Maltworker lung |
| J675 | Mushroom-worker lung |
| J676 | Maple-bark-stripper lung |
| J677 | Air-conditioner and humidifier lung |
| J678 | Hypersensitivity pneumonitis due to other organic dusts |
| J679 | Hypersensitivity pneumonitis due to unspecified organic dust |
| J680 | Bronchitis and pneumonitis due to chemicals, gases, fumes and vapours |
| J684 | Chronic respiratory conditions due to chemicals, gases, fumes and vapours |
| J701 | Chronic and other pulmonary manifestations due to radiation |
| J703 | Chronic drug-induced interstitial lung disorders |
| J704 | Drug-induced interstitial lung disorders, unspecified |
| J708 | Respiratory conditions due to other specified external agents |
| J709 | Respiratory conditions due to unspecified external agent |
| **Rheumatoid arthritis and other inflammatory polyarthropathies** | |
| M050 | Felty's syndrome, unspecified site |
| M051 | Rheumatoid lung disease |
| M052 | Rheumatoid vasculitis |
| M053 | Rheumatoid arthritis with involvement of other organs and systems |
| M061 | Adult-onset Still's disease |
| M064 | Inflammatory polyarthropathy |
| M068 | Other specified rheumatoid arthritis |
| M069 | Rheumatoid arthritis, unspecified |
| M120 | Chronic postrheumatic arthropathy |
| M130 | Polyarthritis, unspecified |
| **Systemic connective tissue disorder** | |
| M321 | Systemic lupus erythematosus, organ or system involvement unspecified |
| M328 | Other forms of systemic lupus erythematosus |
| M329 | Systemic lupus erythematosus, unspecified |
| M331 | Other dermatomyositis |
| M332 | Polymyositis |
| M339 | Dermatopolymyositis, unspecified |
| M340 | Progressive systemic sclerosis |
| M341 | CR(E)ST syndrome |
| M342 | Systemic scleroderma due to drugs and chemicals |
| M348 | Other forms of systemic sclerosis |
| M349 | Systemic sclerosis, unspecified |
| M350 | Sicca syndrome [Sjögren] |
| M351 | Other overlap syndromes |
| M352 | Behçet disease |
| M353 | Polymyalgia rheumatica |
| M354 | Diffuse (eosinophilic) fasciitis |
| M355 | Multifocal fibrosclerosis |
| M356 | Relapsing panniculitis |
| M357 | Hypermobility syndrome |
| M358 | Other specified systemic involvement of connective tissue |
| M359 | Systemic involvement of connective tissue, unspecified |

ATC, anatomical therapeutic chemical classification; ICD-10, International Classification of Diseases 10^th^ revision.

**Table S3. Identification codes for algorithm 3**

| **CCAM code** | **Imaging and respiratory function tests** |
| --- | --- |
| EQQP002 | Measurement of walking distance on level ground in 6 minutes, with monitoring of oxygen saturation by transcutaneous measurement and measurement of useful oxygen flow |
| EQQP003 | Measurement of walking distance on level ground in 6 minutes, with monitoring of oxygen saturation by transcutaneous measurement |
| GLHF001 | Arterial blood sampling with blood gas and pH measurement, without hyperoxia test |
| GLHF002 | Arterial blood sample with blood gas and pH measurement, with hyperoxia test |
| GLQD001 | Measurement of the pulmonary transfer capacity of carbon monoxide (TLCO) or another gas in apnoea or in stable state, during a functional respiratory test |
| GLQP012 | Measurement of slow vital capacity and forced expiration, with recording [Standard spirometry] |
| GLQP002 | Measurement of slow vital capacity and forced expiration, with measurement of lung volumes that can/cannot be mobilised by plethysmography |
| GLQP008 | Measurement of slow vital capacity and forced expiration, with arterial blood gasometry (standard spirometry with blood gas) |
| GLQP009 | Measurement of vital capacity and tidal volume by inductance plethysmography |
| GLQP003 | Forced expiration measurement (flow-volume curve) with recording |
| ZBQK001 | Computed tomography scan of the chest, without intravenous injection of contrast product |
| ZBQH001 | Computed tomography scan of the chest, with intravenous injection of contrast product |
| **ATC code** | **Glucocorticoids and immunosuppressive therapies** |
| L04AA06 | Mycophenolic acid or mycophenolate mofetil |
| L04AX01 | Azathioprine |
| L04AD01 | Cyclosporine |
| L04AD02 | Tacrolimus |
| H02AB02 | Dexamethasone |
| H02AB04 | Methylprednisolone |
| H02AB06 | Prednisolone |
| H02AB07 | Prednisone |
| H02AB09 | Hydrocortisone |
| H02AB10 | Cortisone |
| **GHM code** | **Palliative care** |
| 23Z02T | Palliative care, with or without act, very short duration |
| 23Z02Z | Palliative care, with or without act |
| 23Z03Z | Comfort and other interventions not supported  through compulsory health insurance |
| Z51.5 | Palliative care |
| **LPP code** | **Oxygen therapy** |
| 1118324 | Long-term oxygen therapy, ambulation, Philips, Simplygo |
| 1120338 | Long-term oxygen therapy, ambulation, Invacare |
| 1125100 | Long-term oxygen therapy, concentrator, Scaleo |
| 1130220 | Intensive long-term or ambulatory oxygen therapy, liquid oxygen |
| 1135392 | Oxygen therapy, AVF, weekly service |
| 1136581 | Long-term fixed-station oxygen therapy |
| 1143983 | Long-term oxygen therapy, ambulation, Invacare |
| 1145723 | Hyperinsufflations or in-exsufflations and long-term oxygen therapy |
| 1148130 | Long-term oxygen therapy, concentrator, Invacare |
| 1165967 | Oxygen therapy, AVF, delivery package |
| 1184315 | Long-term oxygen therapy, ambulation, Philips, Ultrafil |
| 1188885 | Hyperinsufflations or in-exsufflations and long-term oxygen therapy fixed station |
| 1191568 | Long-term oxygen therapy, ambulation, Invacare SOL O2 |
| **CCAM code** | **Lung transplant** |
| GFEA005 | Lung lobe transplantation, by thoracotomy without CEC |
| GFEA002 | Lung lobe transplantation, by thoracotomy with CEC |
| GFEA003 | Transplantation of a lung, by thoracotomy without CEC |
| GFEA007 | Transplantation of a lung, by thoracotomy with CEC |
| GFEA004 | Sequential transplantation of the two lungs, by thoracotomy without CEC |
| DZEA004 | Transplantation of heart-lung block, by thoracotomy with CEC |
| **ICD-10 code** | **Hospitalisations for respiratory problems** |
| J* | Disease of the respiratory system |

ATC, anatomical therapeutic chemical classification; CCAM, classification commune des actes médicaux [medical classification for clinical procedures]; CEC, circulation extra-corporelle [extra-corporeal circulation]; GHM, groupes homogènes de maladies [Homogeneous Group of Patients]; ICD-10, International Classification of Diseases 10^th^ revision; LPP, liste des produits et prestations [list of products and services].

**Supplementary Results**

**Table S4. Incidence and prevalence from 2010 to 2016**

|  | **Year** | | | | | | |
| --- | --- | --- | --- | --- | --- | --- | --- |
|  | **2010** | **2011** | **2012** | **2013** | **2014** | **2015** | **2016** |
| **Incidence rate per 100,000 person-years** | 3.96  (3.77–4.17) | 4.12  (3.92–4.34) | 4.43  (4.22–4.65) | 4.63  (4.42–4.85) | 4.73  (4.52–4.95) | 4.63  (4.42–4.85) | 4.59  (4.38–4.81) |
| **Male** | 3.21  (2.99–3.45) | 3.25  (3.03–3.49) | 3.73  (3.49–3.98) | 3.83  (3.59–4.08 | 3.75  (3.52–4.00) | 3.85  (3.61–4.10) | 3.83  (3.59–4.08) |
| **Female** | 2.73  (2.53–2.94) | 2.93  (2.73–3.15) | 2.95  (2.75–3.17) | 3.16  (2.95–3.38) | 3.39  (3.17–3.61) | 3.16  (2.95–3.38) | 3.13  (2.93–3.35) |
| **Prevalence estimate per 100,000 persons** | 6.63  (6.37–6.90) | 9.67  (9.36–9.99) | 12.41  (12.06–12.77) | 14.75  (14.37–15.14) | 16.71  (16.31–17.13) | 18.24  (17.82–18.66) | 19.40  (18.97–19.84) |
| **Male** | 5.15  (4.87–5.44) | 7.44  (7.10–7.79) | 9.71  (9.32–10.11) | 11.52  (11.10–11.95) | 12.76  (12.32–13.21) | 13.99  (13.53–14.46) | 14.85  (14.37–15.33) |
| **Female** | 4.78  (4.52–5.05) | 7.04  (6.72–7.37) | 8.95  (8.59–9.32) | 10.70  (10.30–11.10) | 12.44  (12.02–12.87) | 13.54  (13.11–13.99) | 14.48  (14.03–14.94) |

**Table S5. Overall survival rate by sex**

Overall survival was defined as the time in years from the date of progression to the date of death due to any cause. The cut-off date was fixed at 31 December 2017.

CI, confidence interval; OS, overall survival; PF-ILD, progressive fibrosing interstitial lung disease.

|  | **PF-ILD**  **(n=14,413)** | | |
| --- | --- | --- | --- |
|  | **All**  **(n=14,413)** | **Male**  **(n=7,479)** | **Female**  **(n=6,934)** |
| **Median OS, years (95% CI)** | 3.7 (3.6–3.8) | 3.0 (2.8–3.2) | 4.6 (4.3–4.8) |
| **OS rate, % (95% CI)** |  |  |  |
| 1 year | 73.7 (73.0–74.5) | 70.2 (69.2–71.3) | 77.4 (76.4–78.4) |
| 2 years | 63.8 (62.9–64.6) | 59.2 (58.0–60.4) | 68.6 (67.5–69.8) |
| 3 years | 55.1 (54.2–56.0) | 49.9 (48.6–51.2) | 60.6 (59.3–61.9) |
| 4 years | 48.0 (47.0–48.9) | 42.6 (41.2–43.9) | 53.6 (52.2–55.0) |
| 5 years | 42.0 (41.0–43.0) | 36.5 (35.1–37.9) | 47.8 (46.3–49.3) |
| 6 years | 38.3 (37.2–39.4) | 32.6 (31.1–34.0) | 44.4 (42.8–46.0) |
| 7 years | 34.5 (33.3–35.7) | 29.1 (27.6–30.7) | 40.1 (38.4–41.8) |
| 8 years | 31.6 (30.3–32.9) | 26.4 (24.6–28.2) | 37.1 (35.1–39.0) |

**Table S6. Overall survival rate by age**

Overall survival was defined as the time in years from the date of progression to the date of death due to any cause. The cut-off date was fixed at 31 December 2017.

CI, confidence interval; OS, overall survival; PF-ILD, progressive fibrosing interstitial lung disease.

|  | **PF-ILD**  **(n=14,413)** | | | | |
| --- | --- | --- | --- | --- | --- |
|  | **All**  **(n=14,413)** | **≥20–<50**  **(n=1,890)** | **≥50–<60 (n=1,781)** | **≥60–<75 (n=4,766)** | **≥75–<104 (n=5,976)** |
| **Median OS, years (95% CI)** | 3.7 (3.6–3.8) | – | – | 3.8 (3.6–4.0) | 2.1 (2.0–2.2) |
| **OS rate, % (95% CI)** |  |  |  |  |  |
| 1 year | 73.7 (73.0–74.5) | 89.5 (88.0–90.8) | 83.5 (81.7–85.2) | 74.7 (73.4–75.9) | 64.6 (63.3–65.9) |
| 2 years | 63.8 (62.9–64.6) | 84.2 (82.4–85.9) | 78.0 (75.9–79.9) | 65.0 (63.6–66.5) | 51.1 (49.7–52.5) |
| 3 years | 55.1 (54.2–56.0) | 80.0 (78.0–81.9) | 71.5 (69.2–73.7) | 56.4 (54.8–58.0) | 39.8 (38.4–41.3) |
| 4 years | 48.0 (47.0–48.9) | 76.1 (73.9–78.2) | 66.7 (64.2–69.1) | 48.7 (47.0–50.4) | 30.7 (29.2–32.2) |
| 5 years | 42.0 (41.0–43.0) | 72.0 (69.5–74.3) | 63.0 (60.3–65.6) | 41.8 (40.0–43.6) | 23.7 (22.2–25.2) |
| 6 years | 38.3 (37.2–39.4) | 69.6 (67.0–72.1) | 60.2 (57.3–62.9) | 37.2 (35.3–39.1) | 19.5 (18.0–21.1) |
| 7 years | 34.5 (33.3–35.7) | 66.9 (63.9–69.6) | 56.2 (53.0–59.4) | 33.0 (30.9–35.1) | 15.2 (13.6–16.8) |
| 8 years | 31.6 (30.3–32.9) | 64.0 (60.4–67.4) | 55.1 (51.7–58.4) | 29.3 (27.0–31.8) | 12.0 (10.1–14.0) |

**Table S7. Overall survival rate by diagnosis subgroups**

|  | **PF-ILD**  **(n=14,413)** | | | | | | | | |
| --- | --- | --- | --- | --- | --- | --- | --- | --- | --- |
|  | **All**  **(n=14,413)** | **HP (n=728)** | **IIP (n=3,113)** | **RA-ILD (n=2,521)** | **SSc-ILD (n=907)** | **MCTD-ILD (n=655)** | **Sarcoidosis-ILD (n=1,500)** | **Other autoimmune (n=1,503)** | **Exposure-related ILD other than HP (n=3,486)** |
| **Median OS, years (95% CI)** | 3.7  (3.6–3.8) | 4.8  (4.3–6.0) | 3.7  (3.3–4.1) | 3.5  (3.3–3.8) | 3.1  (2.8–3.6) | 3.6  (2.9–4.2) | 7.9  (7.0–NR) | 6.4  (5.2–7.0) | 2.4  (2.3–2.6) |
| **OS rate, % (95% CI)** |  |  |  |  |  |  |  |  |  |
| 1 year | 73.7  (73.0–74.5) | 81.4  (78.3–84.1) | 70.7  (69.0–72.3) | 72.4  (70.5–74.2) | 73.6  (70.5–76.5) | 71.5  (67.8–74.9) | 85.2  (83.2–86.9) | 81.0  (78.9–83.0) | 67.7  (66.1–69.3) |
| 2 years | 63.8  (62.9–64.6) | 72.8  (69.2–76.1) | 61.1  (59.3–62.9) | 63.0  (61.0–65.0) | 61.5  (57.9–64.8) | 61.7  (57.6–65.6) | 78.4  (76.1–80.5) | 73.1  (70.6–75.4) | 54.7  (52.9–56.5) |
| 3 years | 55.1  (54.2–56.0) | 65.4  (61.4–69.1) | 53.6  (51.6–55.5) | 54.6  (52.3–56.7) | 51.2  (47.4–54.8) | 53.2  (48.8–57.5) | 70.7  (68.0–73.1) | 64.4  (61.7–67.1) | 44.6  (42.7–46.5) |
| 4 years | 48.0  (47.0–48.9) | 57.6  (53.2–61.8) | 48.6  (46.5–50.6) | 46.3  (43.9–48.6) | 43.7  (39.8–47.6) | 45.8  (41.1–50.4) | 64.3  (61.4–67.0) | 59.0  (56.0–61.8) | 35.3  (33.3–37.2) |
| 5 years | 42.0  (41.0–43.0) | 49.2  (44.4–53.9) | 44.3  (42.2–46.5) | 39.8  (37.3–42.3) | 38.8  (34.8–42.9) | 41.1  (36.2–46.0) | 59.0  (55.9–61.9) | 53.6  (50.5–56.7) | 27.8  (25.8–29.7) |
| 6 years | 38.3  (37.2–39.4) | 44.9  (39.7–49.9) | 40.4  (38.1–42.7) | 36.2  (33.6–38.8) | 35.0  (30.7–39.3) | 38.9  (33.7–44.0) | 55.2  (52.0–58.4) | 50.4  (47.0–53.6) | 23.6  (21.7–25.7) |
| 7 years | 34.5  (33.3–35.7) | 40.8  (35.0–46.6) | 36.5  (34.0–39.0) | 31.9  (29.1–34.7) | 29.8  (25.2–34.6) | 30.8  (25.0–36.8) | 53.2  (49.8–56.5) | 46.1  (42.3–49.9) | 20.2  (18.1–22.3) |
| 8 years | 31.6  (30.3–32.9) | 39.6  (33.5–45.6) | 33.8  (31.0–36.7) | 28.9  (25.8–32.1) | 26.7  (21.3–32.4) | 28.7  (22.7–35.0) | 49.5  (45.3–53.6) | 44.6  (40.5–48.6) | 17.3  (15.0–19.8) |

Overall survival was defined as the time in years from the date of progression to the date of death due to any cause. The cut-off date was fixed at 31 December 2017.

CI, confidence interval; HP, hypersensitivity pneumonitis; IIP, idiopathic interstitial pneumonia; ILD, interstitial lung disease; MCTD, mixed connective tissue disease; NR, not reached; OS, overall survival; PF-ILD, progressive fibrosing interstitial lung disease; RA, rheumatoid arthritis; SSc, systemic sclerosis.

**Table S8. Crude multivariable Cox model showing factors associated with mortality**

| **Parameter** | **Reference** | **HR (95% CI)** | **p-value** |
| --- | --- | --- | --- |
| **Sex** | Female | 1.0 |  |
|  | Male | 1.4 (1.31–1.44) | <0.0001 |
| **Categorised age^1^** | ≥20–<50 | 1.0 |  |
|  | ≥50–<60 | 1.5 (1.3–1.6) | <0.0001 |
|  | ≥60–<75 | 2.7 (2.4–3.0) | <0.0001 |
|  | ≥75–<104 | 4.3 (3.9–4.7) | <0.0001 |
| **Type of PF-ILD^2^** | HP | 1.0 |  |
|  | Sarcoidosis-ILD | 0.8 (0.67–0.9) | <0.0001 |
|  | Other autoimmune | 1.0 (0.8–1.1) | <0.0001 |
|  | IIP | 1.4 (1.2–1.6) | <0.0001 |
|  | RA-ILD | 1.4 (1.2–1.6) | <0.0001 |
|  | MCTD-ILD | 1.4 (1.2–1.7) | <0.0001 |
|  | SSc-ILD | 1.5 (1.3–1.7) | <0.0001 |
|  | Exposure-related ILD other than HP | 1.9 (1.6–2.1) | <0.0001 |

Proportional hazards assumption was not respected for the type of progressive fibrosing ILD; therefore, a piecewise Cox model was used (the follow-up time was cut at 12 months).

^1^Age categories were each compared with the 20–50 years age group.

^2^Diagnosis subgroups were each compared with the HP subgroup.

CI, confidence interval; HP, hypersensitivity pneumonitis; HR, hazard ratio; IIP, idiopathic interstitial pneumonia; ILD, interstitial lung disease; MCTD, mixed connective tissue disease; PF-ILD, progressive fibrosing interstitial lung disease; RA, rheumatoid arthritis; SSc, systemic sclerosis.
